# Nine Letters with Answers

**Published:** 1873-01

**Authors:** 


### Our Correspondence.

### LETTERS EXPOSING HUMBUGS AND SWINDLERS,

WITH OTHER COMMUNICATIONS, OF INTEREST TO THE PEOPLE.

MILROY, Pa., Oct. 30, 1872.

THAD. S. UP DE GRAFF, M. D.,.

*Dear Sir:*—Enclosed find a circular of a traveling Dr., bearing the same name as yourself. Let us know, in your next issue of the Bistoury, who he is, or if you know anything of him. He is now at our county seat. (Lewistown). Yours, &c., A. S. H.

We know nothing of him—nor do we care to. We have so repeatedly warned our readers against traveling doctors, whether they bear our name or not, that we imagine but few will be swindled by their deceptions.

---

CLEVELAND O., Oct. 11, 1872.

EDITOR BISTOURY:

*Dear Sir:*—Enclosed please find 50c. for your journal which is ever welcome with its fund of wit, sarcasm, and good advice.

I have suffered for years with what is known as "Hay Fever." I have found relief for this season, if not a permanent cure, in the use of "Crude Petroleum Oil," applied to the nose both externally and internally. When I began using it, I was incapacitated for business. I was relieved in a very few days, and have had no "relapse." If, through the medium of the Bistoury, you can relieve one sufferer, you will certainly have the reward of his gratitude, and win a constant subscriber. Yours, truly,

JNO. E. COLBY.

Mr. Colby's remedy has the merit of being harmless, if not effective. We have learned of several cases that have been similarly relieved.

---

GORHAM, N. Y., Nov. 30, 1872.

EDITOR BISTOURY:

My mother, aged 60, has suffered considerably from inflammatory rheumatism for past year; and now, her eyes have become painful, sensative to the light, and she is nearly blind. She is too sick to be moved to your Eye Infirmary, therefore, what can be done for her?

J. D. S.

Have your family physician prescribe for her rheumatism, and apply to her eyes, with a camel's hair brush, three times a day, the following collyrium:

℞

Atropia sul., gr. vj;

Aquæ rosæ, f. ℥ j. M.

When she is able to get out, have her seen by an ophthalmic surgeon.

---

McKEES, HALF FALLS, Pa., Dec. 2, 1872.

DR. UP DE GRAFF:

I am 30 yrs. old, and have a bunch growing just under my jaw, midway between my chin and ear. It is becoming painful, and sometimes I am quite dizzy. What had I better do about it? P. R.

If it is an abscess of the parotid gland, have it opened. If an enlargement of the gland, or if a tumor, is forming on or near it, have it extirpated. You must consult a competent surgeon, who can at once decide the matter for you.

---

HANCOCK, N. Y., Nov. 20, 1872.

DR. UP DE GRAFF:

*Dear Sir:*—We have a little girl, six-year old, who has had from birth, a drooping of the upper eye lid, with inability to raise it. Physicians have told us that she would out-grow it, but it seems to grow worse. She has no trouble with vision, nor pain in the eye or lid. Can you tell us what the trouble is, and what should be done for it? M——

Your child has what is known to ophthalmic surgeons as *ptosis*, which is a paralysis of the muscle that raises the upper lid. Sometimes curable by administration of tonics of quinia and iron, stimulating embrocations and electricity. Should these means fail, apply to an ophthalmic surgeon and have an eliptical piece removed from the lid, of sufficient size to raise the lid the required distance to remove the deformity.

---

HIESVILLE, KY., Nov. 4, 1872.

EDITOR BISTOURY:

My father, aged 65, has been blind four years, from what the Doctors here call *cataract*. He became gradually blind, without pain or inflammation in his eyes. He is in good health, and of course very anxious to see. A traveling doctor called upon him the other day and induced him to believe that he could "cure the catarac" with roots and herbs. He gave him a gallon or two of decoctions to take "inwardly," and a black salve to smear over his eye-lids. The only change observable is an enormous enlargement of the pupils, which enables him to see more light and thus encourages him to think that the Doctor is doing him good. What I desire to know is whether a cataract *can* be absorbed in this manner, and why the pupils should be so enlarged? J. K. P.

Your traveling quack has used extract of belladonna to enlarge the pupil. It affords more light as long as it is applied, but exerts no influence upon the cataract. It *cannot* be absorbed, and the more of the quack's nostrums he swallows, the worse it will be for him. Take him to a competent oculist and have the cataract removed in the proper manner.

---

QUINCY, ILL., Dec. 9, 1872.

THAD. S. UP DE GRAFF, M. D:

For about one month I have been troubled with a dimness of vision on the outer, or temporal side of my right eye. I am unable to see an object when held to the right of that eye, but when in front, can see as well as ever. One half of my eye seems dark to me, and the black spot seems to increase. I have no pain nor inflammation in the eye, and my general health is good. I have been using my eyes in book-keeping, by artificial light some, perhaps that may be the cause. What should be done, to restore my vision? T. B. S.

Your trouble may arise from a rupture of a small retinal vessel, causing an effusion of blood under the retina. You should consult an ophthalmic surgeon without delay, who can discover the exact condition of affairs by means of an ophthalmoscope. Delay is *very* dangerous in your case.

---

LIVERPOOL, Pa., Dec. 6, 1872.

DR. UP DE GRAFF:

I have a child, one year old, with double hare-lip. When should it be operated upon? B. P.

Now. You have waited too long already, if it is a healthy child.

---

IPSWICH, MASS., Nov., 4, 1872.

EDITOR BISTOURY:

What do you think of "Wyatt's consumption cure?" * * * * * L——

It is a catchpenny concern, to swindle noodles, like you.

